# Supplementary material for: Gastroprotective Effects of Ganoderma lucidum Polysaccharides with Different Molecular Weights on Ethanol-Induced Acute Gastric Injury in Rats
Source: Nutrients. 2022 Apr 1;14(7):1476. doi: 10.3390/nu14071476 (PMC9002462; doi:10.3390/nu14071476)
Supplement: Supplementary file 1 [file nutrients-14-01476-s001.zip › nutrients-1624221-supplementary.pdf]

Table S1. Ulcer index and gastric protection rate（n=10）

Group	Ulcer index（$\bar{X} \pm SD$）	Ulcer inhibition rate（%）
Blank	0	100%
Model	28.37±9.41	0^{a}
Positive	12.73±3.40	55.13^{b}
GLP100-H	11.92±2.33	57.98^{c}
GLP100-M	$12.53±5.10^{b}$	55.83^{b}
GLP100-L	15.62±4.76	44.94^{b}
GLP10-H	14.56±3.37	48.68^{b}
GLP10-M	13.93±4.11	50.90^{b}
GLP10-L	12.21±4.77	56.97^{c}
GLP1-H	15.21±5.23	46.39^{b}
GLP1-M	16.32±7.73	42.47^{b}
GLP1-L	15.27±6.39	46.17^{b}

Different lowercase letters indicate significant differences among treatments ($P < 0.05$).

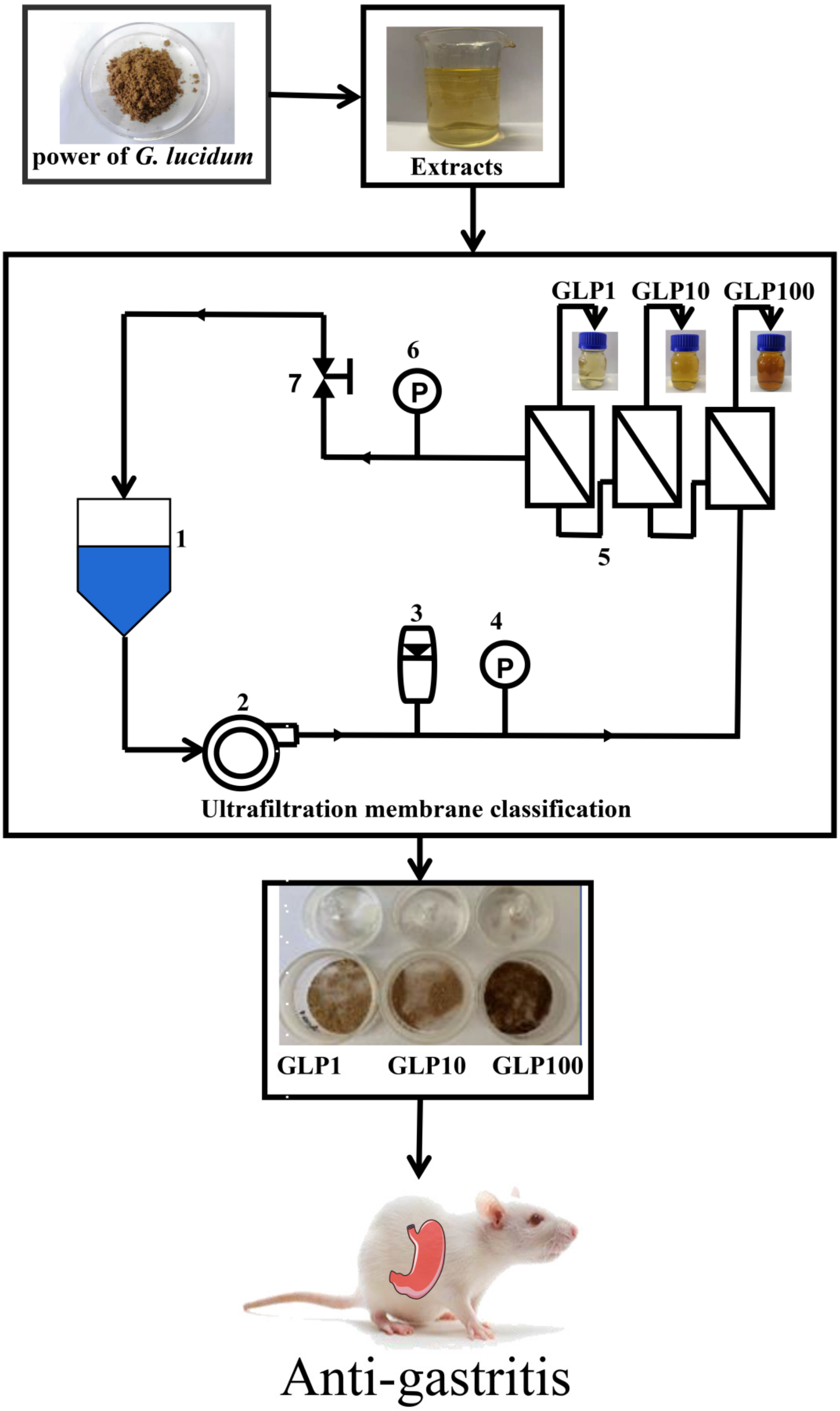

Figure S1. Membrane fractionation and anti-gastritic activities of GLPs

Note: (1) feed tank (2) pump (3) flowmeter (4, 6) pressure gauge (5) membrane module (7) feed valve
